# The construction of a genome‐wide transposon library of *Glaesserella parasuis* and its use to identify genes essential for *in vitro* and *in vivo* survival

**DOI:** 10.1002/mlf2.70106

**Published:** 2026-10-26

**Authors:** Xiaowei Gong, Yuqian Shi, Liuqing He, Qingpo Cui, Guanzheng Zhao, Shuo Xu, Luyu Ying, Qin Yang, Xiaoxia Qin, Tong Sun, Xiaotong Jiang, Juanjuan Wang, Zhangqi Shen, Mengjiao Fu

**Affiliations:** ^1^ State Key Laboratory of Veterinary Public Health and Safety, College of Veterinary Medicine China Agricultural University Beijing China; ^2^ Key Laboratory of Animal Epidemiology of Ministry of Agriculture and Rural Affairs, College of Veterinary Medicine China Agricultural University Beijing China

**Keywords:** essential genes, *Glaesserella parasuis*, Tn‐seq, transposon mutagenesis library, virulence

## Abstract

*Glaesserella parasuis* is an important porcine pathogen that commonly colonizes the upper respiratory tract of pigs and is the etiological agent of Glässer's disease, causing substantial economic losses in the global swine industry. This study aimed to identify genes essential for both *in vitro* growth and *in vivo* pathogenicity of *G. parasuis*. We generated the most comprehensive transposon mutant library in *G. parasuis* to date, containing ~180,000 mutants in the serovar 4 strain H16SD2BY4, with a high TA‐site saturation of approximately 85%. This represents a successful application of transposon insertion sequencing (Tn‐seq) to define essential genes in *G. parasuis* and to screen for genes potentially associated with virulence using a mouse infection model. *In vitro* passaging assays confirmed the exceptional stability of the transposon mutant library and identified 435 genes essential for *in vitro* growth. A genome‐wide screen identified 74 genes specifically required for *in vivo* growth. Among these genes, 14 successfully constructed mutants all showed varying degrees of virulence attenuation in the mouse infection model, confirming the reliability of the transposon library. Additionally, four mutant strains (∆*serA*, ∆*guaB*, ∆*aroE*, and ∆*dgkA*) showed significantly attenuated virulence, highlighting their important roles in *G. parasuis* pathogenesis. The transposon mutagenesis approach used in this work addresses a key gap in analyzing gene essentiality for *G. parasuis* and provides valuable new insights into its pathogenesis.

## Impact statement

*Glaesserella parasuis* is an opportunistic pathogen that causes serious economic losses to the global swine industry. We constructed a *mariner*‐based transposon mutant library in *G. parasuis*, featuring high genome coverage, robust saturation, strong stability, and efficient plasmid elimination. The virulence‐associated genes identified in this screen, including both known and novel virulence‐associated candidates, showed significantly attenuated pathogenicity in mice after deletion, further validating the reliability of our transposon library. This study established an efficient mutagenesis approach and a comprehensive evaluation system, providing a powerful tool for investigating gene essentiality and virulence in *G. parasuis*, and serving as a robust reference for quality assessment of other transposon libraries.

## INTRODUCTION

*Glaesserella parasuis*, a Gram‐negative extracellular bacterium belonging to the *Pasteurellaceae* family, is an opportunistic pathogen that colonizes the upper respiratory tract of pigs^1^. Under appropriate conditions, it can cause Glässer's disease, characterized by fibrinous polyserositis, arthritis, meningitis, pneumonia, and septicemia^2^. The prevalence of *G. parasuis* in pig farms has been reported to range from 8% to 28.54%, causing serious economic losses in the global swine industry^3^, ^4^. Given the impact of *G. parasuis* on the swine industry, gaining a deeper understanding of its pathogenesis is essential.

*G. parasuis* comprises 15 serovars, ranging in virulence from highly virulent to non‐virulent^5^. Serovar 4, considered moderately virulent, is the most prevalent serovar identified in diseased pigs worldwide^6^. However, no clear correlation exists between virulence and serovars. To date, a limited number of virulence factors have been identified in *G. parasuis*, including capsule, lipooligosaccharide (LOS), outer membrane proteins, fimbria, cytolethal distending toxin, and immunoglobulin A protease^7^. To uncover additional potential virulence genes, various approaches have been used, such as comparative transcriptomic and proteomic analyses, as well as genome‐wide association studies, primarily through comparisons between virulent and avirulent strains^8^, ^9^, ^10^. Additionally, RNA‐seq has provided new insights into the possible mechanisms underlying the interaction between *G. parasuis* and host cells during infection^11^, ^12^. However, the low throughput and redundant omics information have limited their efficacy in comprehensively screening *G. parasuis* virulence factors.

Transposon insertion sequencing (Tn‐seq) has risen in popularity for uncovering novel genotype–phenotype relationships in bacteria, as it enables genome‐wide functional screening within a single experiment. Tn‐seq has been proven to be highly effective in identifying virulence factors under specific conditions across a broad range of bacteria, leading to the discovery of over 2000 novel virulence and fitness determinants^13^, ^14^, ^15^, ^16^, ^17^. However, stringent restriction‐modification systems have highly constrained the construction of transposon mutagenesis libraries in *G. parasuis* ^18^. To date, only one Tn*5*‐based transposon mutant library has been constructed in *G. parasuis*. With approximately 10,500 mutants disrupting about 1561 genes, it covers roughly 69% of the predicted 2249 genes in the genome. This limited saturation poses a significant constraint on the comprehensive analysis of gene essentiality and the investigation of virulence factors^19^.

To address this gap, we selected a highly electrocompetent *G. parasuis* strain H16SD2BY4 (serovar 4) and successfully generated a highly saturated transposon mutagenesis library using a *mariner* transposon delivery vector. We comprehensively evaluated the quality of this transposon mutant library to guarantee the reliability and reproducibility of subsequent experiments. This work provides a genome‐wide definition of essential genes in *G. parasuis* under both *in vitro* and *in vivo* conditions. Virulence‐associated genes were identified via Tn‐seq during mouse infection and validated by constructing single‐gene deletion mutants. Our study provides a reliable method for screening virulence factors in *G. parasuis* and establishes a solid foundation for the quality evaluation of other transposon libraries.

## RESULTS

### Construction and assessment of *G. parasuis* transposon mutagenesis libraries

Plasmid pMarG5 is a 5198 bp temperature‐sensitive shuttle vector carrying the *mariner*‐based TnYLB‐1 transposon, including the *Himar1* C9 transposase gene, along with its promoter (Figure S1). Using the well‐characterized *mariner*/*Himar1* transposon, we generated a total of ~180,000 mutants of *G. parasuis* H16SD2BY4 by electroporation, following the workflow shown in Figure S2. All 21 randomly selected colonies showed successful transposon insertion into the genome of H16SD2BY4, with plasmid elimination confirmed (Figure S3). These mutants showed distinct insertion sites, as listed in Table S1.

The *G. parasuis* H16SD2BY4 genome contains 2304 genes and 166,215 TA sites. Over 20 million reads from the transposon mutagenesis library (P0) were aligned to the *G. parasuis* H16SD2BY4 genome, with more than 94% containing the correct transposon tag, achieving ~78‐fold coverage relative to the number of genes in the genome. This identified ~140,000 unique transposon mutants, with an average of one insertion site for every 17 bp (Table S2). The TA‐site insertion saturation of P0 was about 85%. Insertions were distributed throughout the whole chromosome, with no particular hotspots (Figures 1 and S5A). The insertion counts per TA site ranged from 0 to 5530. The rRNA, non‐coding region, and the coding sequence (CDS) of H16SD2BY4 showed the highest average TA density, indicating no specific transposon preference for distinct genomic regions (Figure S4). The carriage rate of the pMarG5 plasmid in three replicates was 0.46%, 0.43%, and 0.42%, respectively.

**Figure 1:**
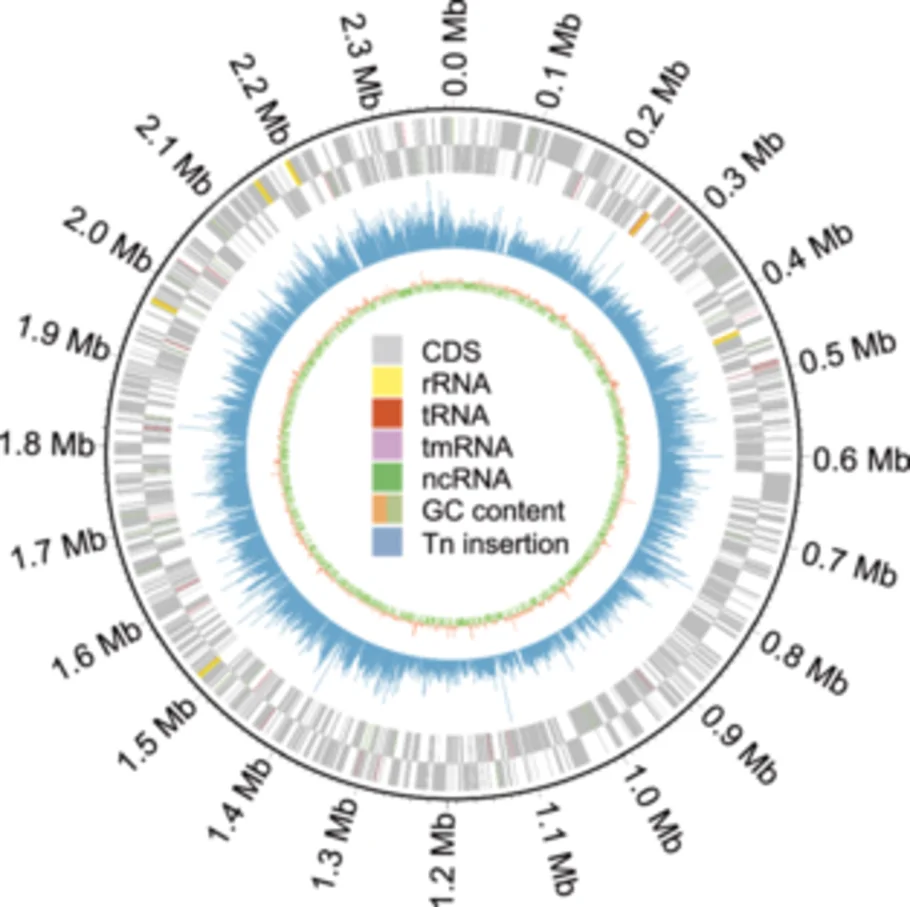
Visualization of the *Glaesserella parasuis* chromosome displaying transposon insertion counts in the input library. Tracks from inside to outside: (1) GC content (orange represents above average GC content and green represents below average GC content); (2) insertion count plot, with the height of the blue bars representing the number of transposon insertions at each TA site (minimum = 0, maximum = 5530); (3) annotated information on the forward and reverse strands (gray, yellow, red, purple, and green represent CDS, rRNA, tRNA, tmRNA, and ncRNA, respectively); and (4) chromosome backbone with ruler. The plot was generated using Circos. CDS, coding sequence; ncRNA, non‐coding RNA; rRNA, ribosomal RNA; tRNA, transfer RNA; tmRNA, transfer‐messenger RNA.

The quality of the transposon input library (P0) was assessed using a saturation curve (Figure S5B). As insertions increased, the number of unique insertion sites plateaued, indicating saturation and sufficient coverage of transposon‐inserted strains. A total of 794 genes showed TA‐site insertion frequencies exceeding 98%, with approximately 85% of genes having more than 50% TA sites disrupted. Notably, 26% of genes had insertions at all of their TA sites (Figure S5C). The three biological replicates of P0 showed strong positive correlations (*r* > 0.97; *R* ^2^ ≥ 0.95) (Figure S5D).

### Gene essentiality analysis of *G. parasuis in vitro*

A total of 435 genes were identified as essential in all three replicate input libraries (P0) by the TRANSIT2 pipeline. The insertion counts of essential genes form a sharp peak at very low values, with the vast majority (97.93%) of these genes harboring fewer than 30 total insertions (Figure 2A). Notably, 92.18% of essential genes had ≤10 insertions and 62.07% had ≤1 insertion, indicating that most essential genes accumulate little to no transposon insertions. In striking contrast, only 4 non‐essential genes (0.65%) had fewer than 30 insertions, establishing a clear and quantitative separation between the two gene classes. Non‐essential genes showed a broad, right‐shifted distribution, with most having 100–250 insertions. The number of essential genes increased progressively from 435 in P0 to 701 in P10 following 10 consecutive passages *in vitro*, while the saturation decreased initially but stabilized after P5 (Figure 2B). The UpSet plot revealed that 431 genes were conserved across all passages (Figure 2C).

**Figure 2:**
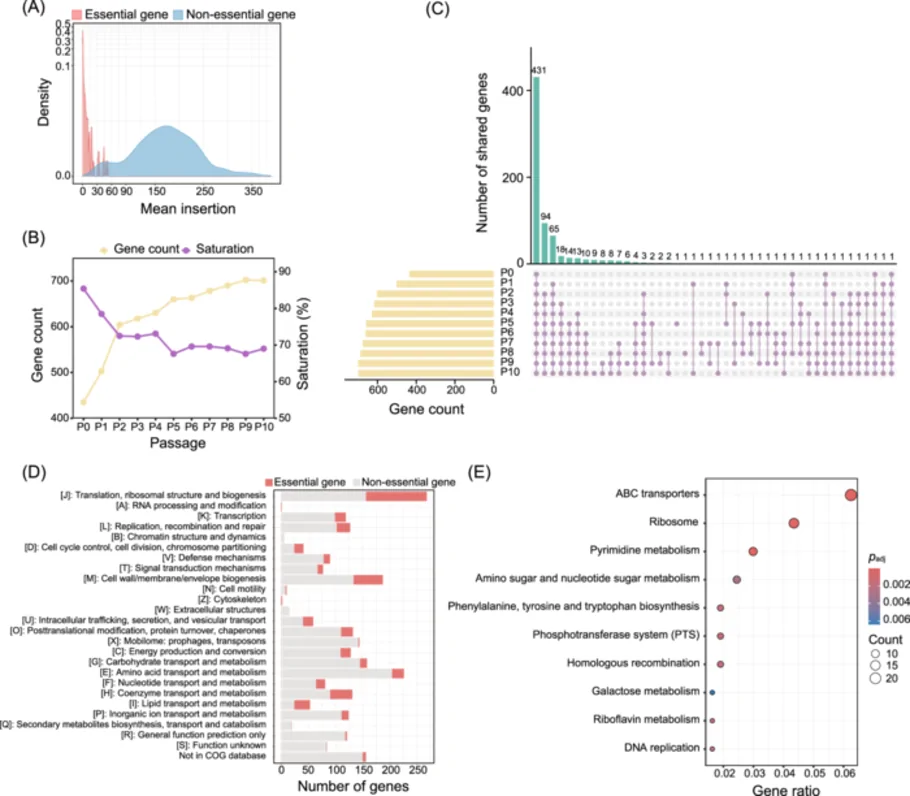
Characterization of 435 essential genes required for *in vitro* survival. (A) Mean insertions of essential genes (pink) and non‐essential genes (blue). (B) Dynamics of gene count (yellow) and saturation (purple) across 10 passages (P0–P10). Gene count represents the number of essential genes required *in vitro* identified in each mutant library. (C) UpSet plot of overlapping genes among 10 passages. The horizontal yellow bar on the left represents the gene count in each passage. The vertical histogram at the top and the dot chart at the bottom together illustrate the number of shared genes across different passages. (D) COG functional classifications of genes in *G. parasuis* H16SD2BY4. Essential genes and non‐essential genes are shown in pink and gray, respectively. (E) Top 10 enriched Kyoto Encyclopedia of Genes and Genomes (KEGG) pathways of essential genes. The color and size of the bubbles represent the adjusted *p‐*value and gene count, respectively. COG, Cluster of Orthologous Groups.

Of the 435 genes classified as essential in P0, 429 were assigned to functional categories in Cluster of Orthologous Groups (COG). The top three classes of essential genes were closely related to the growth of *G. parasuis*, namely, translation, ribosomal structure, and biogenesis (25.7%, 112/435), cell wall/membrane/envelope biogenesis (12.4%, 54/435), and coenzyme transport and metabolism (9.4%, 41/435) (Figure 2D). Gene Ontology (GO) analysis revealed significant enrichment in biological processes (BPs) related to nucleic acid metabolism, tetrapyrrole metabolism, and porphyrin‐containing compound metabolic processes (Figure S6A). Ribosome‐related genes were highly enriched in cellular components (CCs), while ligases, transferases, and DNA helicases were significantly enriched in molecular functions (MFs) (Figure S6A). Kyoto Encyclopedia of Genes and Genomes (KEGG) analysis revealed that the top five enriched pathways were ABC transporters, ribosome, pyrimidine metabolism, amino sugar and nucleotide sugar metabolism, and phenylalanine, tyrosine, and tryptophan biosynthesis (Figure 2E).

### Gene essentiality analysis of *G. parasuis in vivo*

To identify genes essential for *in vivo* survival, the transposon input library (P0) was passaged to P1 (marked as P1‐in below to distinguish it from P1 *in vitro*), followed by infecting mice with a lethal dose of 1.8 × 10^9^ colony‐forming unit (CFU) via an intraperitoneal (i.p.) injection. Samples from different sites including blood, liver, spleen, lung, and brain were harvested and homogenized at multiple time points post infection. A rapid clearance of bacteria from systemic sites throughout the body was observed 3 h post infection (hpi), particularly in the blood and the brain (Figure S7). Approximately 10^7^ to 10^9^ CFU from each site were collected at 3 hpi and pooled to generate output libraries. The TA‐site saturation of the output libraries ranged from 46.3% to 65.3% (Table S2 and Figure S8A). The average number of essential genes required for *in vivo* survival exceeded 600, with 653 identified in the liver, 633 in the spleen, 654 in the lung, 652 in the blood, and 654 in the brain (Figure S8B). Notably, a total of 675 essential genes required *in vivo* were identified, with 620 genes being shared across all five sites (Figure S8C), whereas site‐specific essential genes were relatively rare. These 675 essential genes largely encompassed all *in vitro* essential genes, with 434 and 580 genes overlapping with P0 and P1‐in, respectively (Figure S8D).

### Identification of conditionally essential genes associated with *in vivo* survival

Normalized read counts per locus in each site‐specific output library were compared with those in the input library (P1‐in). To identify genes with significant differences between input and output libraries, a cutoff of an adjusted *p* < 0.05 and a log_2_FC < –2 or >2, corresponding to the ratio of output to input read counts, was imposed. Using these thresholds, 56, 40, 55, 64, and 61 downregulated genes were defined as conditionally essential in the liver, spleen, lung, blood, and brain, respectively (Table S3). Of these, a total of 74 genes were identified as significantly different (excluding essential genes required for *in vitro* P1), with 34 genes shared across all five sites (Figure 3A,B). The major enriched COG classes shifted toward cell wall/membrane/envelope biogenesis (25.7%, 19/74), nucleotide transport and metabolism (16.2%, 12/74), and energy production and conversion (10.8%, 8/74) (Figure 3C). Genes involved in peptidoglycan biosynthesis were associated with top‐ranked GO terms and KEGG pathways (Figures S6B and 3D).

**Figure 3:**
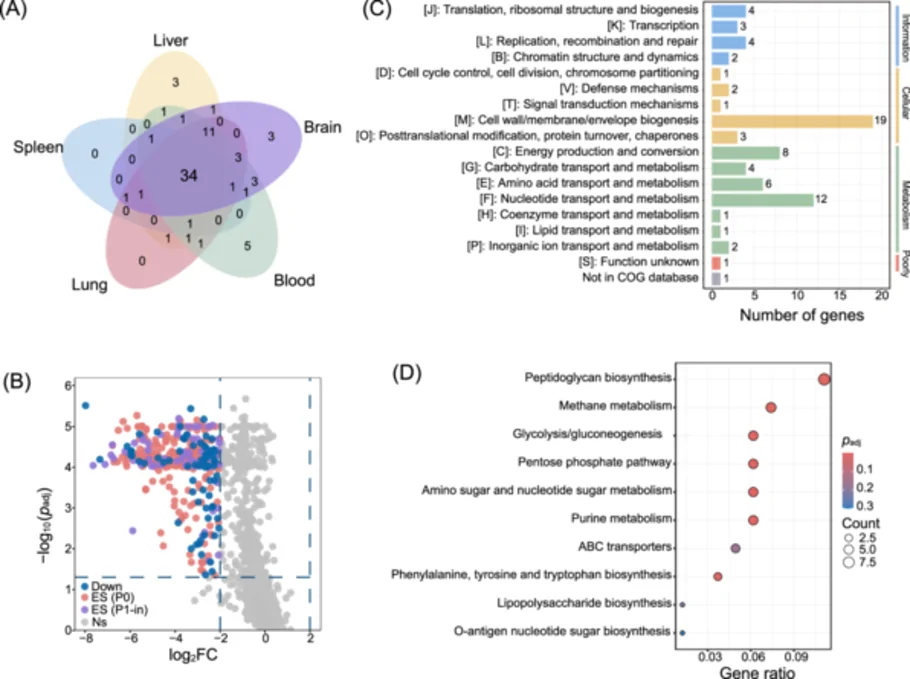
Genome‐wide comparison identifying 74 differential genes between input and output libraries. (A) Venn diagram of 74 differential genes in output libraries across five sites. (B) Comparative analysis of insertion frequency between the input library and output libraries. A significant fitness defect is defined as log_2_FC < –2 and adjusted *p* < 0.05 relative to the frequency of insertions in the input library (blue dots). Genes previously classified as essential in P0 and P1‐in are highlighted in pink and purple, respectively. Gray dots indicate genes with no significant change. Blue dashed lines mark the thresholds for significant gene changes. (C) COG functional classification of 74 differential genes. The bars in different colors denote distinct COG functional categories. The number of genes of each category is indicated at the right of each bar. (D) Top 10 enriched KEGG pathways of 74 differential genes. The color and size of the bubbles represent the adjusted *p‐*value and gene count, respectively.

### Validation of the transposon mutant screen

To validate the screen, we focused on the genes listed in Table S3 and generated single‐gene deletion mutants by replacing each gene of interest with a kanamycin resistance cassette. Finally, we successfully constructed 14 targeted gene deletion mutants via natural transformation, as highlighted in Table S3. No significant differences in growth kinetics were observed among these strains, except for ∆*gpt* and ∆*pgi*, which showed noticeably slower growth compared to the wild type (WT) strain (Figures 4A and S9A). We further evaluated the impact of these mutant genes on virulence using the BALB/c mouse systemic infection model by an i.p. injection of 1.8 × 10^9^ CFU. The survival rates were significantly higher in mice infected with ∆*serA*, ∆*guaB*, ∆*aroE*, ∆*dgkA*, ∆*pgi*, and ∆*gpt* compared to those infected with the WT (*p* < 0.01) (Figures 4B and S9B). However, ∆*pgi* and ∆*gpt* were excluded from further investigation, as their compromised growth kinetics may confound virulence‐specific interpretations. The significant low frequency of transposon insertions in *guaB*, *serA*, *aroE,* and *dgkA* during *in vivo* screening is shown in Figure 4C–F. Notably, *guaB* showed a high |log_2_FC| value of 5.95 (Figure 4C).

**Figure 4:**
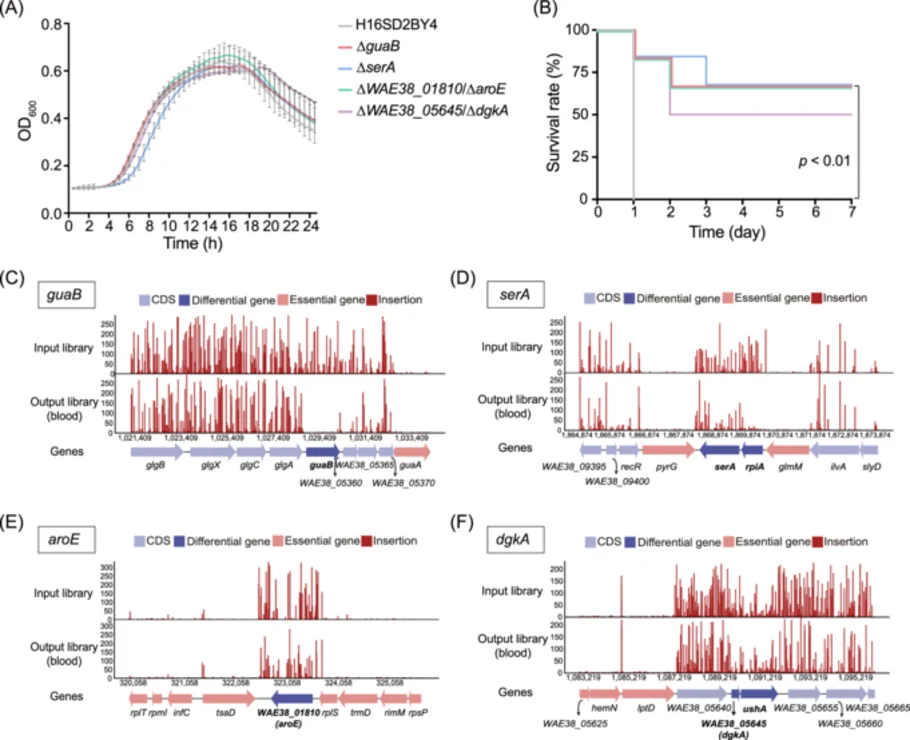
Validation of four potential virulence‐associated genes identified by transposon mutagenesis screen. (A) Growth curves of the wild type (WT, H16SD2BY4), ∆*serA*, ∆*guaB*, ∆*aroE*, and ∆*dgkA*. Data are shown as mean ± SD (*n* = 3). (B) Survival rates of mice infected intraperitoneally with 1.8 × 10^9^ colony‐forming unit (CFU) of the WT, ∆*serA*, ∆*guaB*, ∆*aroE*, and ∆*dgkA* strains (*n* = 6). *p*‐values were determined by the log‐rank (Mantel–Cox) test. (C–F) Comparison of transposon insertion frequencies within the *guaB* (C), *serA* (D), *aroE* (E), and *dgkA* (F) genes between the input library and the output library recovered from blood. The firebrick red bars indicate the normalized transposon insertions at each TA site in the input library (top track) and the output library of the blood (bottom track). Dark purple arrows indicate genes with significantly reduced insertions in the output library (log_2_FC < –2 and an adjusted *p* < 0.05), while light purple arrows represent genes without significant changes. Pink arrows denote genes essential for *in vitro* survival.

## DISCUSSION

We have generated a high‐coverage, high‐saturation transposon mutagenesis library in *G. parasuis* and applied Tn‐seq to identify genes essential for *in vitro* growth and *in vivo* survival. This represents the largest transposon mutant library established in bacteria of the Pasteurellaceae family to date^20^, ^21^. Compared with the previous study, our genome‐scale mutant library provides a key methodological advantage with improved genome coverage, enabling more efficient and reliable identification of essential and fitness‐related genes^19^.

Various attempts were made in this study to optimize the construction of a saturated transposon mutagenesis library in *G. parasuis*. The coverage and saturation of the library largely depend on the electroporation efficiency of the parental strain. In preliminary experiments, we tested multiple strains of different serovars, including three additional serovar 4 strains; however, all strains showed extremely low transposition efficiency (Figure S10A). The type of transposon used may also impact the transposition efficiency. A prior Tn*5*‐derived library of *G. parasuis* serovar 5 strain 29755 contained 10,500 mutants with only 4.67‐fold genomic coverage^19^. Our unsuccessful attempts using Tn*5* in strain H16SD2BY4 yielded only five mutants (Figure S10B). Although the EZ::Tn*5* transposon system is efficient in certain strains, its strain‐dependent activity and high reagent cost make it less suitable for large‐scale library construction. In contrast, plasmid‐mediated transposition systems circumvent these constraints by offering greater operational flexibility and cost‐effectiveness. Additionally, the Tn‐seq amplicon library preparation method is critical for accurate and reliable results. We found that adding a polyC tail to the 3′‐end of transposon junction fragments significantly reduced the genome mapping rate, leading to analysis errors (Figure S10C–D). These methodological refinements are critical for minimizing bias and ensuring reliable downstream analysis.

Transposon library saturation is critical for accurately identifying essential genes. Low‐saturation libraries frequently misclassify dispensable genes as essential due to sparse insertional coverage^22^. Our high‐density library minimizes ambiguity from TA sites lacking insertions, enabling more confident, statistically robust determination of gene essentiality. For essentiality assessment, we used a Hidden Markov Model (HMM), a method validated to perform well even in datasets with insertion densities as low as 20%^23^. Since transposon insertions at coding sequence termini rarely disrupt gene function^13^, ^24^, ^25^, ^26^, we excluded reads falling within the 5% of the 5′‐end and 10% of the 3′‐end. Overall, 435 genes (18.88% of the genome) were identified as essential for *in vitro* survival, consistent with the reported 10% to 25% essential gene proportion across prokaryotes^22^, ^27^, ^28^. Prior work on *G. parasuis* only focused on pan‐genome core genes without systematically screening for essentiality^10^, ^29^. To address this, we performed a comprehensive pan‐genome analysis of 342 *G. parasuis* strains (including H16SD2BY4) using Roary, based on our previously published genomic dataset^6^. This analysis confirmed an open pan‐genome and a core genome of 1308 genes (present in >99% of strains; Figure S11). Notably, 397 of the 435 *in vitro* essential genes (91.26%) belong to this core genome, demonstrating that most genes underlying basic cellular functions under laboratory conditions are highly conserved across the species.

Intrapopulation competition eliminates low‐fitness mutants during passage. As shown in Figure 2, the progressive loss of low‐fitness mutants indicates that some genes contribute to competitive fitness rather than strict viability. The low‐fitness mutants were presumably outcompeted by the higher‐fitness mutants during the growth, ultimately leading to an increase in identified essential genes^30^. This highlights the importance of considering environmental context when interpreting Tn‐seq data. Here, we define genes identified in P0 as strictly essential, genes screened from P0 to P2 as essential under low competition, and genes screened from P2 to P9 as essential under high competition. However, gene essentiality may undergo changes under different selection conditions, underscoring the necessity of *in vitro* passage experiments for screening functional genes under varying pressures (an aspect often overlooked in other transposon library evaluations).

*In vivo* Tn‐seq selection requires isolation of sufficient amounts of bacteria from infected sites to decrease the risk of population bottlenecks^31^. We selected appropriate time points (3 hpi) to retrieve adequate bacterial amounts, thereby avoiding this issue. Overall, 74 differential genes, compared with the input library, were identified as essential for infection. Genes involved in peptidoglycan synthesis accounted for a high proportion and are crucial for maintaining bacterial morphology and protection against environmental stress^32^. Four genes located in the capsule locus of H16SD2BY4 (Table S3) may play a key role in host cell adhesion, immune evasion, and resistance to phagocytosis^33^, ^34^. In addition, *galU*, *galE* ^35^, *lex‐1* ^36^, *rfaD* ^37^, *ompA* ^38^, ^39^, and so forth, were also identified in our study as necessary for the infection and pathogenesis of *G. parasuis*, which confirms the reliability of the transposon mutant library *in vivo*.

To further validate our findings, three distinct gene categories were excluded from subsequent analysis: (i) previously confirmed virulence genes in *G. parasuis*, (ii) capsule/peptidoglycan biosynthesis‐related genes, and (iii) structural protein‐coding elements such as the ATP synthase operon. We successfully generated knockout mutants for 14 genes with previously uncharacterized functions in *G. parasuis*. According to the criteria of Panayidou et al.,^40^ eventually, deletion of four genes, *guaB*, *serA*, *WAE38_01810* (annotated as *aroE* by BLASTn), and *WAE38_05645* (annotated as *dgkA* by Prokka), significantly attenuated the virulence of H16SD2BY4, with no growth defects (Figure 4A,B). The remaining eight mutants (excluding ∆*pgi* and ∆*gpt*) showed mild, statistically non‐significant virulence attenuation. Nevertheless, the potential contributions of these genes to *G. parasuis* virulence cannot be entirely discounted. For example, the periplasmic protease Prc in extraintestinal pathogenic *Escherichia coli* promotes urinary tract infection ability by regulating flagellar expression and bacterial motility^41^. Holmes et al. demonstrated that *purD* is crucial for *Klebsiella pneumoniae* hepatic adaption and serum resistance^42^. Alternatively, the random loss of mutants during *in vivo* infection may have obscured the distinctions between stochastic extinction and true essentiality^43^.

Among the four virulence‐associated genes, *guaB* (encoding IMP dehydrogenase) shows the highest |log_2_FC| value of 5.95. Given the importance of *de novo* guanine biosynthesis for virulence in many bacterial pathogens^44^, ^45^, ^46^, we hypothesize that *de novo* GTP biosynthesis is critical for *G. parasuis* virulence during systemic infection. However, the relative contributions of the salvage versus *de novo* pathways during infection remain to be determined. SerA, identified as a D‐3‐phosphoglycerate dehydrogenase, catalyzes the first step in the phosphorylated pathway of l‐serine biosynthesis^47^. Using transposon mutagenesis, Yasuda M et al.^48^ linked *serA* to adhesion and invasion of *Pseudomonas aeruginosa* PAO1 into Caco‐2 cells, though its mechanism remains uncharacterized. Shikimate dehydrogenase (AroE) catalyzes the conversion of dehydroshikimate into shikimic acid, a crucial precursor for the synthesis of aromatic amino acids. A Tn*5* mutant of *aroE* significantly attenuated virulence in *Xanthomonas oryzae* pv. *oryzae* ^49^, while the *aroE* mutant of *Legionella pneumophila* failed to grow in defined media lacking aromatic amino acids^50^. Given their conserved roles in amino acid biosynthesis and the observed auxotrophic phenotypes of their homolog mutants, we predict that *serA* and *aroE* are auxotrophy‐related genes in *G. parasuis*, a hypothesis requiring experimental confirmation. Collectively, these findings underscore the importance of nutrient acquisition pathways for bacterial virulence. The concept of “nutritional virulence”^51^ may represent an important survival strategy for *G. parasuis* during *in vivo* infection, wherein the bacterium upregulates specific uptake systems to compete for limited host nutrients. Additionally, genes, such as *dgkA*, related to membrane homeostasis may contribute toward maintaining structural integrity under host‐induced stress^52^. In‐depth research on these genes is urgently needed.

We acknowledge several limitations in this study. The use of an intraperitoneal infection model, while necessary for sufficient bacterial recovery, may bypass early colonization processes and overlook genes involved in mucosal infection. Additionally, the stringent bloodstream bottleneck likely eliminates attenuated mutants before organ colonization, leading to underrepresentation of organ‐specific essential genes in our datasets^31^. Attempts to validate findings in the natural host were constrained by reduced virulence of the mutant library and low bacterial recovery, limiting *in vivo* analysis in pigs. Even at a high inoculation dose of 1 × 10^10^ CFU, infected pigs only developed transient hyperthermia without overt clinical signs (Figure S12A–E). Furthermore, mutant yields recovered from pig tissues were too low to construct valid sequencing libraries, indicating that transposon‐mutated strains are highly susceptible to rapid clearance in swine (Figure S12F). Future work should establish a porcine co‐infection or secondary infection model to fully dissect the *in vivo* pathogenic mechanisms of *G. parasuis*.

Construction of a stable transposon library is a prerequisite for screening *in vivo* survival essential genes; yet, no unified standard defines library stability. Our analysis indicated that a proportion of genes identified as essential during mouse infection may also show intrinsic growth defects *in vitro*. Re‐examination of the 74 *in vivo* essential genes showed that 38 were classified as growth defect genes at the fifth *in vitro* passage, and this number increased to 46 in the tenth generation (Table S3). This pattern indicated that continuous *in vitro* passage gradually intensified the competitive selection within the mutant population, gradually depleting low‐fitness strains. Therefore, mutants retained in the early generation libraries for infection experiments may already have weakened competitiveness. Importantly, *in vitro* growth deficiency does not necessarily preclude their roles during infection, as the selection pressure encountered by the host was quite different from that under laboratory culture conditions. These findings highlight the inherent challenges of Tn‐seq screening and suggest that integration of *in vitro* passage data and *in vivo* selection results could improve the interpretation of key pathogenic determinants.

In conclusion, we successfully constructed a *mariner*‐based *Himar1* transposon mutagenesis library (180,000 mutants, 85% TA saturation) for *G. parasuis* and provided a genome‐wide view of genes contributing to bacterial survival and pathogenicity. This work establishes a valuable resource for functional genomics and offers insights into the mechanisms underlying host adaptation. Further characterization of identified candidates will advance our understanding of *G. parasuis* pathogenesis and support the development of targeted intervention strategies.

## MATERIALS AND METHODS

### Mice

Female BALB/c mice aged 6–8 weeks were obtained from SPF (Beijing) Biotechnology Co., Ltd. Mice were acclimatized under standardized environmental conditions (temperature: 23 ± 2°C; humidity: 55% ± 10%) for 1 week before the experiments.

### Bacterial strains and growth conditions

*G. parasuis* strain H16SD2BY4 (GenBank accession No. CP147984), maintained in our laboratory, was grown on TSA or in TSB supplemented with 25 μg/ml nicotinamide adenine dinucleotide and 5% (v/v) fetal bovine serum. When necessary, media were supplemented with kanamycin (50 μg/ml) or gentamycin (10 μg/ml).

### Construction of a temperature‐sensitive shuttle vector

The fragment containing the temperature‐sensitive replication region and the gentamycin‐resistance gene of pCmTn5 (modified from pSHG5 previously in this laboratory) was amplified using primers pSHG5V‐F/R (Table S4). Another fragment containing the *mariner*‐based TnYLB‐1 transposon and the *Himar1* C9 gene along with its promoter was amplified from pMar4s using primers *Himar*‐F/R (Table S4). These two products were fused by overlap extension PCR to construct the pMarG5 plasmid. The resulting plasmid, pMarG5, was confirmed to be as predicted (Figure S1) using primers pSHG5‐F/R (Table S4) and DNA sequencing.

### Generation of the *G. parasuis* transposon mutant library

Electrocompetent cells of *G. parasuis* H16SD2BY4 were prepared as previously described, with slight modifications^53^. Cells from a 100 ml TSB culture in the logarithmic phase of growth were pelleted by centrifugation at 8000*g* for 10 min, washed three times with ice‐cold sucrose/glycerol (SG) buffer (15% [v/v] glycerol, 272 mM sucrose), and resuspended in a final volume of 1.5 ml of SG buffer. Then, cells (100 μl) were mixed with 1 μg of pMarG5 DNA before electroporation at 2.5 kV, 200 Ω, and 25 μF. Bacterial suspensions were cultured at 30°C for 2 h and inoculated onto TSA plates supplemented with 50 μg/ml kanamycin at 42°C for 1–2 days. Positive colonies and transposon insertion sites were verified using primer pairs IRS + Tn‐overlapF/R and primers SP1–SP3, respectively (Table S4). About 180,000 transformants were scraped off the plates and pooled in 30% (v/v) TSB glycerol for storage at –80°C for subsequent experiments.

### Transposon mutant library passage

Approximately 1 × 10^8^ CFU of P0 were inoculated into 100 ml of TSB supplemented with 50 μg/ml kanamycin and incubated at 37°C with shaking for 8 h. Subsequently, 1 ml of the culture was transferred into 100 ml of fresh TSB containing 50 μg/ml kanamycin and cultured under the same conditions for another 8 h. This process was repeated for a total of 10 consecutive passages.

### Transposon library screening in a mouse infection model

The transposon library (Input: P0) was passaged, collected, and CFU was measured on TSA plates. Then, cells were pelleted by centrifugation at 5000*g* for 10 min and washed twice with PBS. BALB/c female mice (*n* = 12 per group) were challenged with a dose of 1.8 × 10^9^ CFU of *G. parasuis* H16SD2BY4 mutants (Input: P1‐in) via an intraperitoneal injection. Blood was collected, mice were euthanized, and organs including the lung, liver, spleen, and brain were removed and homogenized in sterile PBS at 3 hpi. All tissue homogenates and blood were plated onto TSA plates supplemented with 50 μg/ml kanamycin. All colonies (>180,000) from each site were individually scraped from the plates and pooled in TSB containing 30% (v/v) glycerol for storage at –80°C, serving as the output libraries.

### Transposon library sequencing

Upon completion of pooling, bacteria were pelleted, and genomic DNA (gDNA) was extracted. For each library, 1 μg of purified gDNA was fragmented to an average size of ~250 bp using the VAHTS Universal Plus Fragmentation Module (Vazyme) and fragmented DNA was prepared for sequencing using the NEBNext Ultra II DNA Library Prep Kit for Illumina (NEB) for end repair and adaptor ligation. The T‐tailed adaptor was generated with primers Adaptor_Top/Bottom (Table S4) using a PCR thermal procedure (37°C for 11 min and 85°C for 30 min). As recommended by Long et al.^54^, transposon–gDNA junctions were selectively amplified through two rounds of PCR. The first round utilized a short transposon‐specific primer (Transposon_Enrich_Fwd) and an adaptor‐specific primer (Transposon_Enrich_Rev) to amplify and enrich gDNA flanking the insertion site. The second round used a hemi‐nested PCR to add sequences containing barcodes (Nextera_R1/R2, Table S4) required for subsequent Illumina sequencing. The PCR products were cleaned using 0.9 × VAHTS DNA Clean Beads (Vazyme) to remove DNA fragments smaller than 200 bp. The quality and concentration of each sample were assessed using an Agilent 2100 Bioanalyzer (Agilent Technologies) and a Qubit 4.0 Fluorometer (Life Technologies), respectively. The resulting fragment libraries were sequenced on the Illumina HiSeq X Ten platform (Annoroad), obtaining at least 10 million 150‐bp paired‐end reads per sample.

### Sequencing analysis

The elimination of the pMarG5 plasmid was evaluated by a custom Python script, seq_statistic.py. Reads were processed and mapped to *G. parasuis* H16SD2BY4 chromosome using the TPP software tool in the TRANSIT2 pipeline (https://transit.readthedocs.io/en/latest/)^23^. HMM was applied to TPP‐derived insertion counts to predict the most probable essentiality status for each TA site and annotated gene. The insertion counts in each sample were normalized by Total Trimmed Read‐count (TTR). To calculate gene essentiality, read counts located in the 5′‐end (5%) and the 3′‐end (10%) of genes were filtered out, as many essential genes appear to tolerate insertions in these regions^55^, ^56^. Essential genes identified in all three input libraries (P0 and P1‐in) or in all three output libraries for each site were separately extracted to generate lists of genes essential for *in vitro* or *in vivo* survival, respectively. To compare differences in gene read counts between the output (*in vivo*) libraries and the input library, the Mann–Whitney *U*‐test in TRANSIT2 was applied, using TTR normalization for standardization. The *p* values were adjusted using the Benjamini–Hochberg method. Genes with an adjusted *p* value below 0.05 and an absolute log_2_ (fold change) (|log_2_FC|) greater than 2 were identified as statistically significant. Reproducibility was evaluated by calculating Pearson's correlation coefficient (*r*) for the ratio of TA sites with insertions per gene to the total TA sites per gene. Pairwise correlation coefficients (*R* ^2^) are indicated using R v4.4.1.

### Functional annotation analysis

For functional annotation analysis, COG, GO, and KEGG Orthology numbers for the genes of interest were obtained by eggnog v4.5.1 (https://github.com/eggnogdb/eggnog-mapper)^57^. Enrichment analyses for COG (https://ftp.ncbi.nlm.nih.gov/pub/COG/), GO (https://geneontology.org/), and KEGG pathway (https://www.kegg.jp/brite/br08611) were performed using the custom Python and R scripts. An enrichment significance level of *p* ≤ 0.05 was used as the threshold.

### Construction of *G. parasuis* gene deletion mutants

The primers used for amplification in this study are listed in Table S4. The upstream and downstream fragments adjacent to the deleted gene were amplified from H16SD2BY4 gDNA using two primer pairs (primers with prefixes P1–P4), and the kanamycin cassette was amplified from pSHK4 using primers with prefixes P5 and P6. These three PCR products were purified and fused by overlap extension PCR using primers with P1 and P4 prefixes. Notably, a 9‐bp core DNA uptake signal sequence (USS) was incorporated into all homologous arm constructs^58^. The resulting fragment was subsequently cloned into linearized pK18mobsacB to generate the recombination plasmid. The recombinant plasmid was introduced into *G. parasuis* H16SD2BY4 by natural transformation, as described previously^58^. Positive transformants were verified by PCR with primers prefixed with P11–P14 and sequencing.

### Growth curve assay

A single colony of WT (H16SD2BY4) and mutant strains was individually picked and inoculated into 1 ml of TSB and grown overnight at 37°C. Then, the suspension was adjusted to a final concentration of approximately 1.0 × 10^6^ CFU/ml and inoculated into a 96‐well microplate, with three replicate wells per strain. The OD_600_ was measured every 30 min with continuous shaking for 24 h at 37°C by an Infinite M200 Microplate reader (Tecan) in the long‐term model. The experiment was performed with three biological replicates.

### Survival curve assay

The WT and mutant strains were individually inoculated into 100 ml of TSB and cultured to the logarithmic growth phase. Cells were then pelleted by centrifugation at 5000*g* for 10 min and washed twice with PBS. Mice were intraperitoneally infected with these strains at a dose of 1.8 × 10^9^ CFU and monitored daily for 7 days.

### Statistical analyses

The statistical analysis was conducted in GraphPad Prism 10 software. *p*‐values were calculated using an unpaired *t* test between two groups or one‐way ANOVA among multiple groups. Survival curves were assessed by the log‐rank (Mantel–Cox) test.

## AUTHOR CONTRIBUTIONS

**Xiaowei Gong**: Data curation; formal analysis; investigation; methodology; software; validation; visualization; writing—original draft; writing—review and editing. **Yuqian Shi**: Investigation; methodology; validation; writing—review and editing. **Liuqing He**: Investigation. **Qingpo Cui**: Software. **Guanzheng Zhao**: Investigation; methodology. **Shuo Xu**: Investigation. **Luyu Ying**: Investigation. **Qin Yang**: Investigation; methodology. **Xiaoxia Qin**: Investigation; methodology. **Tong Sun**: Investigation. **Xiaotong Jiang**: Investigation. **Juanjuan Wang**: Supervision. **Zhangqi Shen**: Conceptualization; funding acquisition; project administration; resources; supervision; writing—review and editing. **Mengjiao Fu**: Conceptualization; resources; supervision; writing—review and editing.

## ETHICS STATEMENT

The animal experiment protocol was reviewed and approved by the Animal Ethics Committee of China Agricultural University (No. AW30905202‐2‐01), and all procedures were carried out strictly in accordance with the relevant regulations and guidelines issued by this ethics committee.

## CONFLICT OF INTERESTS

The authors declare no conflict of interests.

## Supporting information

Supporting file 1.

Supporting file 2.

Supporting file 3.

Supporting file 4.

Supporting file 5.

## Data Availability

The sequencing raw data are available in the GenBank database under the BioProject accession number: PRJNA1206794.
