# Supplementary material for: The construction of a genome‐wide transposon library of Glaesserella parasuis and its use to identify genes essential for in vitro and in vivo survival
Source: mLife. 2026 Oct 26;5(5):626–36. doi: 10.1002/mlf2.70106 (PMC13710409; doi:10.1002/mlf2.70106)
Supplement: Supplementary file 1 [file MLF2-5-626-s002.docx]

**Supporting information**

**Figure S1. Construction of a temperature-sensitive shuttle vector for pMarG5.** The fragment containing temperature-sensitive replication region and gentamycin-resistance gene of pCmTn5 was amplified. Another fragment containing *mariner*-based TnYLB-1 transposon was amplified from pMar4s. These two fragments were combined to construct pMarG5 plasmid by fusion PCR.

**Figure S2. Schematic workflow for constructing transposon mutant libraries in *G. parasuis*.** In stage 1, pMarG5 is introduced into *G. parasuis* H16SD2BY4 by electroporation and all transformants plated on selective agar plates are collected and pooled as the input library. In stage 2, the input library (P0) is passaged ten consecutive times. In stage 3, the input library is used to challenge mice in a systemic infection model, with organs and blood harvested and processed at 3 h post challenge. In stage 4, genomic DNA is extracted from both input and output libraries, followed by DNA fragmentation, adaptor attachment, barcode amplification, and Illumina sequencing. *In vitro* essential genes were identified via a Hidden Markov Model (HMM); conditionally essential genes for *in vivo* survival were identified by comparing the precise transposon insertion sites between the input and output libraries. Culture media: Tryptic soy agar/broth (TSA/TSB; complex and undefined media), supplemented with 50 μg/mL kanamycin. This diagram was created with Adobe Illustrator 2021.

**Figure S3. Confirmation of transposon insertion and plasmid elimination.** (**A**) Verification of the transposon insertion. (**B**) Verification of the plasmid elimination. Lane M, DNA DL2000 marker ladder; Lane 1-21, colonies selected randomly; Lane +, pMarG5 as positive control; Lane 2B4, H16SD2BY4; Lane -, negative control.

**Figure S4. Distribution of transposon insertions across the genome of H16SD2BY4.** (**A**) Genome composition of H16SD2BY4. (**B**) Average TA density in different genomic regions.

**Figure S5. Assessment of saturation and quality of the input library.** (**A**) Distribution of insertion events in the input library. Insertions are counted every 100 bp. (**B**) Saturation level of the input library. For each point, unique insertion events are calculated among the selected insertion sites. (**C**) Frequency of insertion at TA sites of all genes. (**D**) The reproducibility of the input library among three biological replicates. Each point represents the ratio of TA sites with insertions per gene to the total TA sites per gene. Pairwise correlation coefficients (R^2^) are indicated.

**Figure S6. GO enrichment analysis of 435 essential genes (A) and 74 differential genes (B).** Top 8 enriched terms in BP, CC and MF are shown in green, pink and blue, respectively.

**Figure S7. Bacterial dynamics of H16SD2BY4 in blood and organs.** Bacterial loads in blood (**A**), liver (**B**), spleen (**C**), lung (**D**) and brain (**E**). (**F**) Total survival rate (defined as the ratio of the total bacteria loads to the inoculum) of bacteria at different time points post-infection. Data are shown as mean ± SD (n = 3).

**Figure S8. Characterization of essential genes required for *in vitro* and *in vivo* survival.** (**A**) Saturation of output libraries. Each site has three biological replicates, indicated by the purple dots. (**B**) Number of essential genes required for survival in different sites. (**C**) Venn diagram of essential genes required for survival in five sites. (**D**) Shared essential genes both *in vitro* (P0、P1-in) and *in vivo*.

**Figure S9. Validation of 10 mutant strains identified by transposon mutagenesis screen.** (**A**) Growth curves of the WT and 10 mutant strains. Data are shown as mean ± SD (n = 3). (**B**) Survival rate of mice infected i.p. with 10^9^ CFU of the WT and 10 mutant strains (n = 6). *P*-values were determined by unpaired t test in (A) and the log-rank (Mantel-Cox) test in (B).

**Figure S10. Optimization of transposon mutagenesis and Tn-seq library preparation for *G. parasuis*.** (A) Electroporation efficiency of various *G. parasuis* strains used in preliminary transformation attempts. (B) Five mutants obtained using the Tn*5*-based transposon system in strain H16SD2BY4. (C) Representative raw sequencing reads from the polyC-tailing method for Tn-seq library preparation. The extensive stretches of consecutive G nucleotides that caused reduced genome mapping rates were highlighted with red lines. (D) Mapping and insertion count statistics of transposon mutagenesis libraries constructed using the polyC-tailing method.

**Figure S11. Pangenome summary statistics of 342 *G. parasuis*.** (A) Genome size evolution curves of the pan-genome (blue) and core-genome (pink). (B) Proportion of core, soft-core, shell, and cloud genes.

**Figure S12. *In vivo* characterization of the transposon mutant library (P1-in) compared with the WT strain H16SD2BY4 in a pig infection model.** Pigs were intraperitoneally inoculated with 1 × 10^10^ CFU of the indicated strains. (A) Body temperature of pig inoculated with the WT strain. (B) Canine sitting posture observed in pig inoculated with the WT strain. (C) Bacterial loads in blood (at 10 and 12 hpi), heart, liver, spleen, lung, kidney, brain and ascites of infected with the WT strain. (D) Body temperature of pig inoculated with P1-in. (E) Gross anatomical examination of heart, liver, lung, spleen, kidney and brain from pig inoculated with P1-in. (F) Mapping and insertion count statistics of transposon mutagenesis libraries for the pig experiment.

**Table S1. Detection of transposon insertion sites.** The red font indicates that the transposon inserted the same gene in strain 11 and 13, but at different insertion positions, 787 bp and 338 bp in the direction of the start codon of *WAE38_00730*, respectively.

**Table S2. Mapping and insertion count statistics of transposon mutagenesis libraries.** Matched (%) represents the percentage of reads with valid Tn prefix. Mapped (%) represents the percentage of reads mapped into H16SD2BY4 reference genome. Saturation (%) represents the percentage of TA sites with at least one insertion. NZ Mean represents the mean read count over non-zero TA sites.

**Table S3. Genes required for survival in different sites and the corresponding knockout mutant strains.** Mean log_2_FC was calculated from the average fold-change across the corresponding sites. Gene names that were not annotated in GenBank have been assigned by Prokka v1.12. Genes successfully knocked out in this study are highlighted in pale yellow.

**Table S4. Primers used in this study.** The underline indicates the area of overlap between upstream and downstream. 5PHOS/ represent phosphorylation at the 5'-end. The NNNNNNNNNN represents the indexing barcode used for sequencing and is varied with each sample. The red letters represent the USS.
